# An overview of using qualitative techniques to explore and define estimates of clinically important change on clinical outcome assessments

**DOI:** 10.1186/s41687-019-0100-y

**Published:** 2019-03-04

**Authors:** Hannah Staunton, Tom Willgoss, Linda Nelsen, Claire Burbridge, Kate Sully, Diana Rofail, Rob Arbuckle

**Affiliations:** 1 Roche Products Limited, Hexagon Place, 6 Falcon Way, Shire Park, Welwyn Garden City, AL7 1TW UK; 2 GlaxoSmithKline, Collegeville, PA USA; 3 Clinical Outcomes Solutions, Folkestone, UK; 4 Adelphi Values, Patient-Centered Outcomes, Macclesfield, Cheshire UK

**Keywords:** Important change, Meaningful change thresholds, Responder definition, Concept elicitation, Cognitive debriefing, Exit interviews, Focus groups, Vignettes, Delphi panel

## Abstract

Establishing meaningful change thresholds for Clinical Outcome Assessments (COA) is critical for score interpretation. While anchor- and distribution-based statistical methods are well-established, qualitative approaches are less frequently used. This commentary summarizes and expands on a symposium presented at the International Society for Quality of Life Research (ISOQOL) 2017 annual conference, which provided an overview of qualitative methods that can be used to support understanding of meaningful change thresholds on COAs. Further published literature and additional examples from multiple disease areas which have also qualitatively explored the concept of meaningful change are presented.

Semi-structured interviews conducted independently from a clinical trial, exit interviews conducted in the context of a clinical trial, focus groups, vignettes and the Delphi panel method can be used to obtain data regarding meaningful change thresholds, with advantages and disadvantages to each method. Semi-structured interviews using concept elicitation (CE) or cognitive debriefing (CD) methods conducted independently from a clinical trial can be an efficient way to gain in-depth patient/caregiver insights. However, there can be challenges with reconciling heterogeneous data across diverse samples and in interpreting the qualitative insights in the context of quantitative score changes. Semi-structured qualitative interviews using CE/CD methods embedded as exit interviews in a clinical trial context with patients/caregivers can provide insights which can augment quantitative findings based on analysis of clinical trial data. However, there are logistical challenges relating to embedding the interviews in a clinical trial.

Focus groups and the Delphi panel method can be valuable for reaching consensus regarding meaningful change thresholds; however, for face-to-face interactions, social desirability bias can affect responses. Finally, using vignettes and taking a mixed methods approach can aid in achieving consensus on the minimum score change endorsed by respondents as a meaningful improvement/decrement. However, the approach can be cognitively challenging for participants and reaching a consensus is not guaranteed.

Anchor- and distribution- based methods remain critical in establishing responder definitions. Nonetheless, qualitative data has the potential to provide complementary support that a certain level of change on the target COA, which has been statistically supported, is truly important and meaningful for the target population.

## Introduction

Establishing what constitutes clinically important change on Clinical Outcome Assessments (COAs) is valuable to aid interpretation of COA data by patients, clinicians, regulators and payers [1, 2]. It is increasingly recognized that standard approaches performing anchor-based and distribution-based quantitative analyses can be enhanced by incorporating qualitative methods as part of a patient-centric, mixed methods approach. Such qualitative methods can be used to obtain the perspectives of patients, caregivers and healthcare professionals regarding what constitutes an important score change [3].

Establishing clinically important change on COAs is not a new concept. It has been discussed in the literature with various terminology and definitions proposed at different times [4]. Minimal clinically important difference (MCID) was the first term to be proposed [5] and emphasised the patient perspective in defining the MCID as: *“the smallest difference in score in the domain of interest which patients perceive as beneficial”* [6]*.* Other key terms of note include minimal important difference (MID) [7], broadly defined as the smallest difference that is considered clinically important to patients. This terminology was used in the 2006 Food and Drug Administration (FDA) Patient-Reported Outcome (PRO) draft guidance [8]. The final FDA PRO guidance (2009) [9] and the more recent Patient Focused Drug Development (PFDD) draft guidance series focus on establishing individual-level change (i.e., responder definition) as opposed to differences between groups [10–12].

Given recent emphasis on the use of responder definitions, there is renewed focus on the identification of thresholds that can be considered an ‘important’ amount of change as opposed to ‘minimal’ change [13]. Moreover, the value of exploring the patient perspective, rather than relying purely on anchor/distribution-based methods, is also being increasingly endorsed. Understanding patient, caregiver and clinician perspectives of what constitutes important change is valuable to augment and contextualise responder definitions derived from anchor-based (i.e., exploration of the association between the targeted concept of the COA instrument and the concept measured by the anchor) [9] and distribution-based analyses (i.e., use of the distribution of the COA score to quantify the magnitude of the change) [3]. Conducted properly, qualitative insight allows us to move beyond determining simply that ‘x’ change is important; it allows us to explore how and why it is important, translating this change into real-world improvement.

However, ‘important’ change can be a challenging concept to understand, particularly for patients and caregivers, but also for healthcare professionals. The optimal methods for eliciting this input from patients qualitatively are far from established, and many challenges remain associated with collecting, analysing and interpreting qualitative data related to meaningful change thresholds. Moreover, in the few examples in the literature, a variety of approaches have been used including semi-structured interviews embedded in clinical trials and conducted independently of clinical trials, focus groups, Delphi panels and the presentation of vignettes.

In an effort to build upon recent momentum and interest, this commentary reports on a symposium session at the International Society for Quality of Life Research (ISOQOL) 2017 annual conference. The symposium provided a comprehensive overview of qualitative techniques that can be used to establish important change on COAs with case studies used as illustrative examples. This article intends to summarize and expand on the symposium content by outlining further published literature on this topic, and by providing additional examples from multiple disease areas which have also qualitatively explored the concept of meaningful change.

## Main text

### Overview of the ISOQOL symposium

The symposium session included five presentations and was focused on using qualitative methods to explore and define estimates of clinically meaningful change and responder thresholds, including an outline of advantages, challenges and potential solutions. The first presentation provided an overview of qualitative techniques including potential benefits and limitations. See sections Semi structured interviews conducted externally to a clinical trial to Vignettes (bookmarking/standard setting) for an evaluation of each approach.

The second presentation summarized results from a qualitative exit interview study with psychologists and parents/caregivers to help inform clinically meaningful change thresholds on an observer-reported, clinician- administered measure and a performance outcome measure in Down syndrome. The third and fourth presentations focused on results from qualitative interviews and an associated analysis of clinical trial data in pediatric asthma. The overarching aim of this research was to establish clinically meaningful change in symptom-free days (SFDs), rescue free days (RFDs) and on the childhood Asthma Control Test (C-ACT) in pediatric asthma. The final presentation focused on the use of clinical trial exit interviews to generate anchors for incorporation in later psychometric evaluation. See sections Semi-structured interviews conducted externally to a clinical trial and Semi-structured exit interviews conducted as part of a clinical trial for further detail and elaboration on these examples.

### Overview of qualitative research techniques to define meaningful change

Numerous qualitative methods have been used to capture patient, caregiver and clinician perspectives of important change. These include semi-structured interviews conducted externally to a clinical trial, semi-structured interviews conducted as part of a clinical trial (exit interviews), focus groups, the presentation of vignettes and the Delphi panel method. When considering the conduct of semi-structured interviews, Concept Elicitation (CE) or Cognitive Debriefing (CD) techniques can be used to explore a particular condition or COA measure. As outlined by Patrick et al., (2011) the concept elicitation process starts deliberately broad and open-ended in order to explore and define information, usually about a particular condition, from the perspective of the patient [14]. The cognitive debriefing process on the other hand (also called cognitive interviews) is more structured and usually focused upon a specific COA measure, with direct questions about understanding, relevance and comprehensiveness of the measure. Both of these interview techniques can be used to explore what would potentially be a meaningful change in a condition or on a specific COA measure. Moreover, these methods can be employed in interviews conducted independently of a clinical trial or embedded as part of a clinical trial in order to explore important change on target COAs.

CE questioning within a semi-structured interview can provide non-biased, spontaneous insights into what an important change in symptoms would be, based upon individual experiences. Such qualitative data, even when captured from a sample who are not clinical trial participants, can be used to augment quantitative findings from a clinical trial by providing in-depth qualitative descriptions of the patient experience, for example from patients at various levels of functioning or disease severity. The technique of CD within a semi-structured interview can also help to provide better understanding of thresholds for important improvements/decline in the context of a response scale or score change. This would involve asking patients about their understanding of the underlying scale and then establishing the level of change on the scale that they would consider meaningful.

Other approaches to exploring meaningful change include focus groups, the presentation of clinical vignettes and the Delphi panel method. Although there is a not a fixed procedure for focus groups, they typically involve a group discussion among six to ten participants, with high moderator involvement to guide the discussion and to ensure participants keep to topic and all share their views [15]. Again, CE or CD interview techniques can be used to discuss meaningful change in a focus group setting. The presentation of vignettes, specifically the bookmarking/standard setting method, is another approach which has been used to derive qualitative meaningful change insights. This typically involves presenting patients and/or experts with hypothetical clinical vignettes in order to reach a consensus on thresholds for meaningful change. Finally, the Delphi panel method is a technique which involves using a series of questionnaires or ‘rounds’ to gather perspectives, obtain feedback and reach consensus on a given topic [16]*.*

The remainder of this article evaluates these qualitative techniques for establishing important score change on COAs and provides examples of each approach.

#### Semi-structured interviews conducted externally to a clinical trial

Semi-structured interviews conducted externally to a clinical trial can provide preliminary insights regarding meaningful concepts for a treatment to target, as well as anecdotal insights regarding what level of change in these concepts is important. Moreover, this approach can provide useful insights when it is not possible to incorporate an interview in a clinical trial for logistical reasons. Semi-structured interviews involve the interviewer leading the discussion based on a pre-determined interview guide. However the participant’s responses help guide the level of information generated about the predetermined topics and their relative importance [10]. One challenge of this approach is how to link the qualitative comments to potential score changes on the measure and reconciling heterogeneous qualitative insights, especially for complex, multi-domain COAs. In addition, the target sample’s experience of change in the concept of interest must be considered in the context of the severity of their disease; meaningful change thresholds may differ depending on severity (*see Katz et al. 2015*, for example) [17]. It is also important to ensure that the population interviewed matches the severity of the clinical trial population as the severity of the disease can potentially significantly affect patient’s perception of meaningful change. Moreover, discussing hypothetical change versus being able to reflect upon real changes that patients have experienced may lead to the generation of different evidence. Therefore, exploring change in the context of a real-world setting where actual change could be expected to occur is most beneficial in collecting these insights.

##### Example of an interview study conducted externally to a clinical trial

As discussed during the symposium, a recent study in pediatric asthma involved using both CE and CD techniques when interviewing patients and caregivers in an observational, non-interventional setting [18]. The primary objective of this research was to generate evidence to support estimates of the individual within-patient changes that constitute an important or meaningful change in symptom-free days (SFDs) and rescue-free days (RFDs) and updated estimates on the Childhood Asthma Control Test (C-ACT) in pediatric asthma populations aged 5–11 years.

**Fig. 1:**
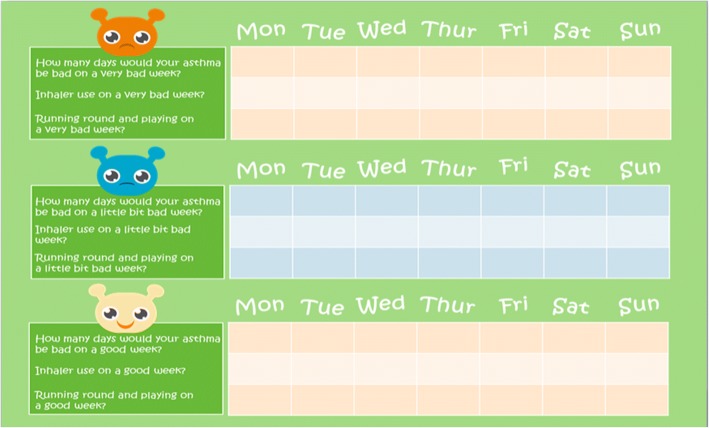
Calendar task

Semi-structured, qualitative interviews were conducted with children (aged 8–11 years old) who had asthma and their parents/caregivers, as well as independent caregivers of 5–7 year olds. Participants were asked to complete diaries before their interview. During the interview the children and caregivers were asked to talk about symptoms they experienced on a ‘good’ day and a ‘bad’ day with their asthma (i.e., use of CE style questions) and whether the difference between ‘good’ and ‘bad’ days was meaningful. Additionally, a calendar task [ Fig. 1] was used to help the children think about the hypothetical number of days that would comprise a “very bad”, “little bad”, and “very good” week with asthma. Finally, using the diary data collected 4–9 days before the study as a starting point for discussion, the interviewer used CD interview techniques and a ‘think aloud’ process to understand changes experienced over the period and whether the participant considered those changes meaningful and important.

Children and parents/caregivers provided generally concordant estimates of the number of days with asthma symptoms in ‘very bad’, ‘little bad’ and ‘very good’ asthma weeks, with the findings suggesting that a change of ≥1–2 days per week is meaningful and important, which was broadly concordant with the findings for RFDs. In addition to being able to discuss “important” change, parents/caregivers were also able to talk about the concept of “minimal” change, suggesting that 1–2 additional SFDs or RFDs would be the smallest meaningful improvement. Both parents/caregivers and children were able to articulate what a meaningful level of change would be on the C-ACT at the item level. This qualitative study generated C-ACT item-level meaningful change estimates in the region of a 1–3 category change.

This study highlights that the use of CE /CD interview techniques in semi-structured interviews conducted independently of a clinical trial can provide valuable insights regarding meaningful change and can represent a useful alternative when embedded exit interviews are not possible. Both children with asthma and their parents/caregivers could qualitatively articulate meaningful change in SFDs and RFDs.

In addition, this qualitative data was triangulated with distribution-based analyses performed using independent clinical trial data. Anchor-based methods were not possible due to a lack of adequately correlated anchors. For SFDs and RFDs the results were similar to the qualitative results (i.e., suggesting changes of 1–2 additional symptom or rescue free days in a seven-day period would be above measurement error), providing support for qualitative data generating enhanced confidence in quantitative results. The C-ACT analyses were based on the total score and ranged from 1.67–4.37 on the total score (0–27). The qualitative data however is not comparable here because the interviews focused on item level meaningful change as discussing the total score was considered too abstract for participants. The challenge of discussing meaningful change on multi-item PROs is perhaps even more pronounced in stand-alone qualitative studies where participants are not familiar with the target COA in the same way they would be in a clinical trial.

#### Semi-structured exit interviews conducted as part of a clinical trial

**Exit or embedded interviews** incorporated as part of a clinical trial are being utilised as a means of obtaining patient perspectives on whether actual changes experienced during a trial are meaningful and important. An advantage of exit interviews is that patients are familiar with the target COA from the clinical trial period and at least some of the patients have likely experienced change of some description over the study course. However, patients may not have insight into how the measure is scored or how a change in the totality of the measure might be relevant. Therefore qualitative discussion may be focused on the concepts measured and small versus large changes, as opposed to dialog surrounding a specific score change. Exit interviews can also be used to obtain patient-derived anchors for change that can be used to supplement analyses using clinical trial anchors and distribution-based statistical techniques [19]. Being embedded within the clinical trial offers the unique opportunity to explore the patient perception of the *same* changes as those that are being evaluated by the clinical trial endpoints, thus increasing interpretability.

There are several recent examples in the literature of exit interviews being used to explore clinically important change across a variety of therapeutic areas [20–22]. Challenges in conducting exit interviews include the potential for feedback obtained in the interview to conflict with clinical trial data, which may make the clear interpretation of study findings more difficult, particularly if the exit interview data highlights a lack of meaningful change from a patient perspective in those where change is observed on COA measures. There are also logistical challenges to consider including adverse event reporting, maintaining data privacy which will vary depending on whether the exit interviews are incorporated into the main trial protocol versus collecting the data in a follow-up study. The optimal timing of the interview in order to minimize recall bias is imperative. The interview should either be embedded in the trial when meaningful change is anticipated to have occurred or be conducted as close to a patient exiting the trial as possible. The discussion during the interview should be explicitly focused upon the trial period to avoid confusion with any other changes either before or after participation in the trial (for example, since discontinuing treatment). Additionally, recall bias can be minimized by prospectively incorporating entry questions/interviews at the start of the trial intervention to act as a ‘reliable’ anchor for areas of focus during the exit interview, and to reduce the bias of retrospective assessment [23].

##### Examples of exit interview studies

As discussed during the symposium, a recent example in Down syndrome (DS) involved using both CE and CD interview techniques with caregivers and expert clinicians in an exit interview context to explore clinically important change [24]. This study aimed to establish, via qualitative exit interviews, the level of change that is clinically important on the Vineland Adaptive Behavior Scales, Second Edition (Vineland™-II) [25] and the Repeatable Battery for the Assessment of Neuropsychological status (RBANS) [26] instruments in a DS population. Psychologists (*n* = 10) and caregivers (*n* = 8) who participated in a clinical trial were interviewed after they had exited the trial.

**Fig. 2:**
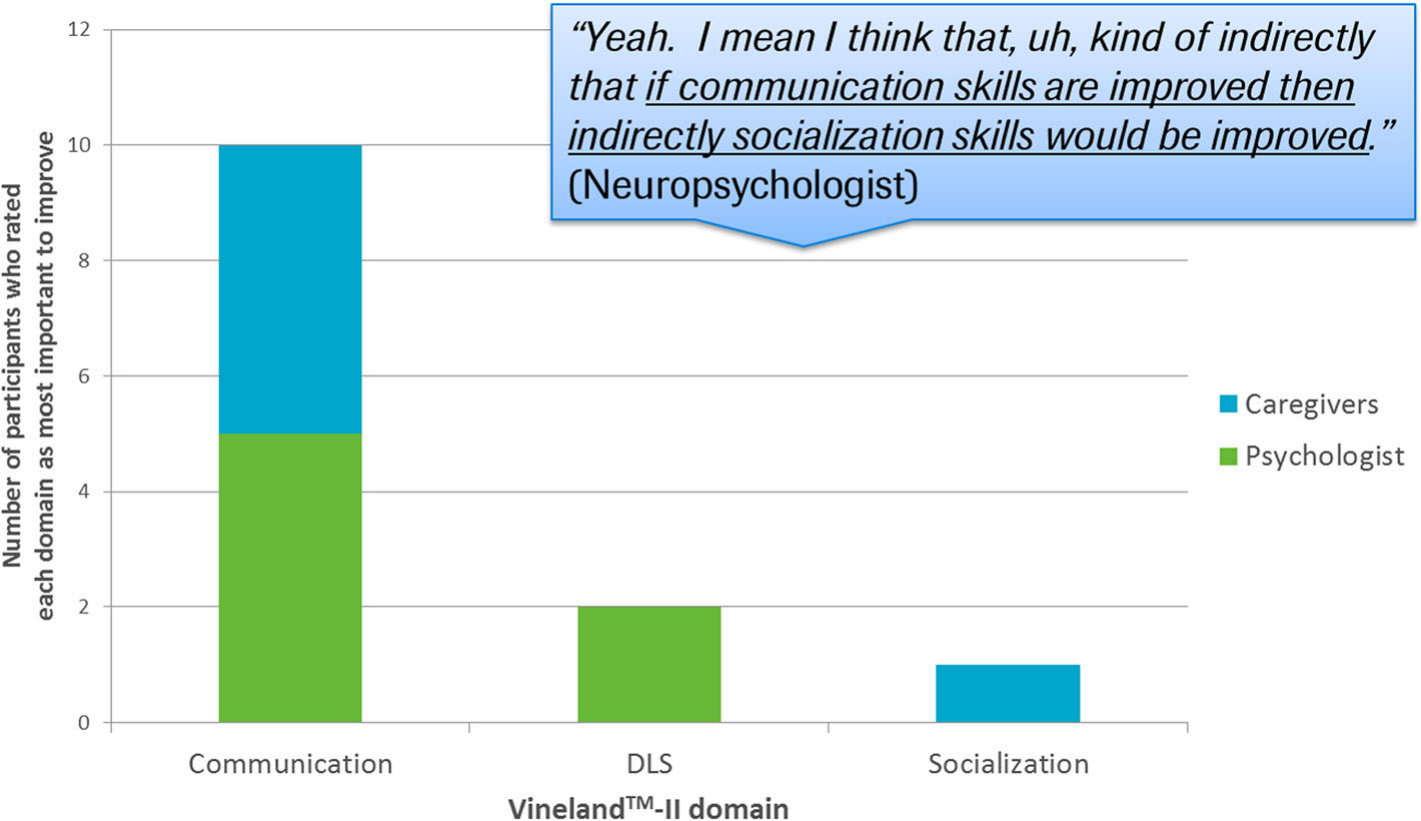
Which Vineland™-II domain is most important to change?

Overall, the study generated rich data and caregivers and psychologists were able to provide detailed insights into which domains/concepts are most important to improve. The communication domain of the Vineland™-II was rated as the most important domain to improve by the majority of participants asked (*n* = 5 caregivers and *n* = 5 psychologists) [ Fig. 2]. While such meaningful change insights were not grounded in score change on the target COA, understanding which concepts are most important to improve from a patient and caregiver perspective can be highly valuable and informative when considering endpoint selection where it is imperative that conceptually relevant and important aspects of a disease are being measured.

Notably, during this study, caregivers generally considered any change to be important (e.g. from not being able to perform an activity at all to doing something sometimes or with assistance) while psychologists provided larger meaningful change estimates. Despite the creative use of CE/CD interview techniques, the caregivers (and psychologists) also sometimes struggled to respond to questions focussed on complex and abstract scales. Therefore, despite caregivers and psychologists being familiar with this scale from the clinical trial, given its complexity, participants still struggled to articulate a meaningful score change on the instrument and this would likely be the case regardless of the qualitative method employed to discuss meaningful change. Future studies may benefit from a domain-level focus or use of vignettes to anchor qualitative responses in order to give respondents something tangible to consider.

Another example is an exit interview study that was conducted following the Xermelo clinical trial with a subset of 35 of the 135 trial participants. The findings indicated that 17 of the exit interview participants identified diarrhea as the single most important symptom to treat, and frequency and urgency was most often selected as the single most important aspect of diarrhea they wished to improve [27]. Moreover, anchor-based data generated during the exit interviews were evaluated alongside anchors included in the full trial sample to determine that the differences between the treatment and placebo groups in the change in frequency of bowel movements observed during the trial were meaningful and important. Thus, the exit interview sub-study provided confirmation that the primary endpoint was important to patients and provided additional anchors to aid interpretation of that endpoint. Exit interviews can therefore be used in multiple ways to better understand the meaningfulness of clinical trial data. More recently there have been a number of exit interview studies published and the field continues to learn about the most optimal approach to conducting and generating valuable insights using this data type [28, 29].

#### Focus groups

**Focus groups** can be a useful forum to discuss important change, where CE or CD techniques can be used. The advantages of focus groups include having the potential to reconcile conflicting data and reach consensus on key aspects of meaningful change that are important to the participants, while also reducing costs and timelines. However, individual differences may mean reaching consensus is challenging and, as in any focus group study, social desirability bias may influence the comments that some participants make. The participant(s) with the strongest opinions may unduly influence other participants and the findings. An alternative that potentially attenuates some of the challenges of face-to-face focus groups is to conduct the group discussion via an online discussion panel. This approach is likely to reduce bias, and also gives participants more time to consider their responses as well as enabling participants to contribute at a convenient time, despite having the potential to increase dropout rates and timelines. A further challenge (as outlined in section Semi structured interviews conducted externally to a clinical trial ) is that if participants have not experienced change, reflection on hypothetical change can be a challenge for participants and potentially less reliable. In addition, lack of familiarity with the target COA can be a barrier to informed discussion.

##### Examples of focus group studies

The use of focus groups to discuss clinically meaningful change is not commonplace in the literature due to a preference for individual semi-structured interviews. However, two studies in rare diseases have used focus groups with success to discuss this topic. The first study in spinal muscular atrophy adopted face-to-face focus groups to explore meaningful change in Type II and non-ambulant type III patient populations, associated with treatment of this condition [30]. Given the progressive nature of the condition, small changes in motor function were considered to have an important impact on quality of life and maintenance of current ability was considered to be a meaningful outcome. The second study, in Duchenne and Becker Muscular Dystrophy involved an online focus group (*n* = 12) as well as interviews (*n* = 4) with individuals and caregivers and an expert committee regarding meaningful outcomes that extend beyond function, using the Pediatric Outcomes Data Collection Instrument (PODCI) as a starting point [31]. Vitality/fatigue; mental health; peer relationships; self-efficacy and autonomy; and a sense of hope and purpose were identified as key themes. The authors concluded that such outcomes measured using Health Related Quality of Life (HRQoL) measures may not be responsive to change. Overall, both of these studies provided very important insights regarding concepts of importance to measure and the ability of outcome measures to detect such changes.

#### Vignettes (bookmarking/standard setting)

**Vignettes** are valuable for exploring meaningful change on a target COA as they provide a starting point for patients to evaluate meaningful change. The benefits of this approach have been noted primarily in relation to the modified bookmarking/standard setting approach [3]. The method does not require an interventional study and ties the threshold directly to the patient perspective, while giving respondents a starting point from which to consider their own symptoms. The rigorous approach advocated by Cook and colleagues requires a relatively large sample to support item response theory (IRT) based quantitative analyses and reaching a consensus on the threshold is not guaranteed [32]. While less robust, if logistical considerations do not allow for such an involved approach there is still value in taking a similar approach but with a smaller sample, using vignettes derived from qualitative sources, and placing more emphasis on the qualitative insights generated. However, irrespective of how the vignette is designed, the approach can be cognitively challenging for patients as it is asking patients to reflect on scenarios where there may be subtle differences between vignettes.

##### Examples of studies employing vignettes

With regards to examples in the literature, clinical vignettes for different severity levels on the NeuroQOL have previously been created from an IRT calibrated bank [32]. In this study patients with multiple sclerosis and experts were recruited. For severity levels associated with lower extremity function and sleep disturbance, patients and experts derived the same threshold. However, for upper extremity function and fatigue there were differences, with patients setting higher thresholds for more severe classifications of symptoms. This approach can also be extended to ask patients to consider their symptoms in context of the vignette and whether they are experiencing symptoms that are greater, the same, or lesser than the vignette description, and to then hypothetically consider a meaningful or important change in the context of the vignette [33]. In order to estimate clinically important improvement/decrement values, differences endorsed as enough to make a difference by > 50% were calculated. This approach was used by Cook and colleagues to establish meaningful change on the NeuroQoL fatigue short form [33]. The use of qualitative vignettes has also been employed in a recent study evaluating clinically meaningful change on a self-reported assessment of ‘worst itch’ in Atopic Dermatitis [34]. During semi-structured interviews three vignettes (as well as qualitative questioning) depicting mild, moderate and severe symptoms were used as a basis for patients to discuss meaningful change. Patients were asked if the itch in each vignette was the same, worse or better than their own current itch and if any differences were meaningful. In addition, patients were asked if there were meaningful differences among the vignettes. The smallest level of change that each patient considered meaningful could then be established. The majority of participants suggested either a two- or a three-point change was the smallest change that would be meaningful. Overall, patients found the vignette task easier to understand than the questions about meaningful change that used qualitative questioning, based on the patient’s own reported score on the numerical response scale.

#### Delphi panel method

Benefits and points for consideration of the Delphi panel method have been outlined in the literature and are generally consistent with a remote focus-group approach [16]. Advantages include the ability to reach a large number of individuals across a variety of locations, and by conducting this anonymously a single individual is less likely to bias the results. However, variability in execution of the Delphi procedure exists, including defining when sufficient consensus has been reached and attrition rates can be high. However, guidance to improve the optimal use and reporting of the Delphi method has been developed [16].

##### Examples of studies employing the Delphi panel method

There are numerous examples of using the Delphi panel method to define meaningful change in the context of clinical trials, most commonly with groups of expert clinicians. [35–37] The Delphi panel method has also been highlighted as a way to reach a consensus on a single MCID, where multiple estimates have been generated [38]. The method has also recently been used to establish meaningful or important change thresholds for PRO measures in the literature with an example in chronic obstructive pulmonary disease [35]. Here physicians were sent material on clinically meaningful change estimates from published literature/patient change scenarios and were asked to outline what they considered to be a minimal, moderate or large amount of clinically meaningful change. During subsequent rounds, additional literature was added and results synthesized. In general, the expert panel meaningful change estimates were larger than previous studies based on patient-reported clinically important differences.

## Discussion/Conclusions

Qualitative methods can generate valuable insights into what constitutes clinically important change on COAs. Regulators, policy makers and industry are increasingly highlighting the value of such data. For example, as part of the PFDD voice of the patient FDA meetings, patients’ perspectives on what an ideal treatment would look like and what clinical benefit would be the most meaningful to them is routinely asked.

Exit interviews embedded as part of a clinical trial are arguably the most effective way to generate qualitative meaningful change insights. This is because a proportion of the sample has potentially experienced change due to the intervention and therefore can reflect on the importance of this. Their comments can then be linked to changes observed during the trial. If it is not possible to embed exit interviews in the context of a clinical trial, vignettes can provide a useful alternative in which to frame a meaningful change discussion outside of a clinical trial setting. Providing participants with a scenario which asks them to consider if their symptoms are greater, the same or lesser than the vignette description can provide a useful reference point for reflection, ultimately helping to drive the discussion and insights gathered. While semi-structured interviews, focus groups or Delphi panel methods conducted externally to a clinical trial can be viable alternatives to exit interviews and the vignette approach, there are more challenges with these methods. However, in the case of the former approaches (semi-structured interviews and focus groups), a meaningful discussion regarding important concepts to measure and relevance to everyday life and proposed outcome measures can be achieved. In addition, the Delphi panel method can and has been used to assist with establishing a threshold where multiple values exist.

It is important to highlight the limitations to this commentary. The first limitation is that this was not a systematic review of all articles published on the use of qualitative methods to establish meaningful change on COAs, and is instead intended as an overview of key techniques with selected examples. Furthermore, with regards to the original research presented (i.e., the DS and pediatric asthma examples) both samples were relatively small and future research would benefit from further considering patient sample size in order to be confident in the meaningful change threshold information gathered. Moreover, this article has not considered all techniques for qualitatively establishing meaningful change. There are additional approaches such as conjoint analysis which warrant further exploration.

When considering directions for future research, the field would benefit from greater consideration of clinically important decline, as the majority of qualitative meaningful change studies, including those conducted in the context of a clinical trial, focus on understanding meaningful or important improvement. Statistically it has been found that the threshold for meaningful worsening may be different from those for meaningful improvements [39]. This can be very important in evaluating interventions for which delaying progression is the goal.

Future research may also benefit from considering novel or more informal sources of qualitative meaningful change information where participants may be more open, such as information gathered from social media which has been used with success when generating the patients’ perspective of their disease experience [40]. Additional guidance from FDA and European Medicines Agency (EMA) on appropriate qualitative methods for the purpose of understanding meaningful or important change and regarding triangulation across qualitative and quantitative methods would be valuable to researchers designing clinical trial endpoints. Of note, exit interviews, cognitive interviews and surveys are cited in the context of helping to inform improvement thresholds in the FDA’s recent draft guidance on collecting comprehensive and representative input for PFDD [11]. Finally, future research should seek to address the limitations of the various qualitative approaches outlined in this article. For example, when considering semi-structured interviews conducted externally to a clinical trial, creative approaches such as incorporating vignettes could be considered to aid the comparison between qualitative comments generated in the interview and potential score changes on COAs. For exit interviews, including this approach in early clinical trials (e.g., phase 1b or II studies) could allow for more efficient adoption and therefore minimize logistical challenges in larger studies. As highlighted in the main body, researchers could also consider entry questions/interviews to minimize recall bias if the trial is of significant duration.

Despite the numerous examples that illustrate the value of collecting qualitative insights in defining important change, it is emphasized that such methods complement and enhance the interpretation of quantitative methods, they are not meant to replace them. Anchor/distribution-based approaches remain critical in estimating a responder definition and anchor-based methods can indirectly provide patient-derived data on meaningful change thresholds (e.g., through use of a patient global impression item). However, while all qualitative methods for estimating important change have their limitations, so too do quantitative methods where the appropriateness of anchors and limitations of specific data sources can also raise questions about generalizability [41]. Triangulating data from multiple sources yields greatest confidence in the appropriateness of both anchors and meaningful change estimate thresholds; the inclusion of qualitative data adds a further valuable perspective [38, 42]. Moreover, there are situations where data from anchor-based methods has considerable limitations, such as in the context of rare diseases, where sample sizes are often limited, or if the anchor(s) available poorly match the target concept [43]. In such situations the use of qualitative methods to understand the perspectives of individual patients, caregivers and clinicians may assume greater importance and can provide highly valuable insights. Qualitative insights can also be particularly valuable when quantitative approaches are limited by anchors which are poorly understood or have weak correlations with the target outcome of interest.

One consistent finding across the case studies presented in this article is that patients and caregivers will often consider very small magnitudes of improvement to still be important. However, this may be unique to the populations studied and could be expected to vary by condition and severity level. It should be recognized that slightly larger magnitudes of improvement will likely be necessary to satisfy regulators, reimbursement authorities and clinicians, in order to ensure that the improvements are real and important and not simply due to chance or ‘noise’ in the measurement. In the context of drug development, the combination of qualitative insights alongside anchor and distribution responder based estimates can aid in setting realistic and clinically meaningful target product profiles in relation to the outcomes of interest. Overall, the optimal approach to establishing a responder definition is one that employs a mixed methods approach which involves the purposeful collection and analyses of a range of both qualitative and quantitative data [44] to fully capture what constitutes a clinically important change on a COA in the specific context of use.

## Abbreviations

C-ACT: Childhood Asthma Control Questionnaire
CD: Cognitive debriefing
CE: Concept elicitation
COA: Clinical Outcome Assessment
DS: Down syndrome
EMA: European Medicines Agency
FDA: Food and Drug Administration
HRQoL: Health Related Quality of Life
IRT: Item response theory
ISOQOL: International Society for Quality of Life
MCID: Minimal clinically important difference
MID: Minimal important difference
PFDD: Patient Focused Drug Development
PODCI: Pediatric Outcomes Data Collection Instrument
PRO: Patient-Reported Outcome
RBANS: Repeatable Battery for the Assessment of Neuropsychological status
RFDs: Rescue-free days
SFDs: Symptom-free days
Vineland™-II: Vineland Adaptive Behavior Scales, Second Edition

## References

[1] Keefe RS, Kraemer HC, Epstein RS (2013). Defining a clinically meaningful effect for the design and interpretation of randomized controlled trials. Innovations in clinical neuroscience.

[2] McLeod LD, Coon CD, Martin SA, Fehnel SE, Hays RD (2011). Interpreting patient-reported outcome results: US FDA guidance and emerging methods. Expert review of pharmacoeconomics & outcomes research.

[3] Coon CD, Cappelleri JC (2016). Interpreting change in scores on patient-reported outcome instruments. Therapeutic Innovation & Regulatory Science.

[4] King MT (2011). A point of minimal important difference (MID): A critique of terminology and methods. Expert review of pharmacoeconomics & outcomes research..

[5] Guyatt G, Walter S, Norman G (1987). Measuring change over time: Assessing the usefulness of evaluative instruments. J Chronic Dis.

[6] Jaeschke R, Singer J, Guyatt GH (1989). Measurement of health status: Ascertaining the minimal clinically important difference. Control Clin Trials.

[7] Guyatt, G. H., Osoba, D., Wu, A. W., Wyrwich, K. W., Norman, G. R., & Clinical Significance Consensus Meeting Group. (2002, April). Methods to explain the clinical significance of health status measures. *Mayo Clinic Proceedings, 77*(4), 371-383. 10.4065/77.4.371 11936935

[8] U.S. Department of Health and Human Services FDA Center for Drug Evaluation and Research, U.S. Department of Health and Human Services FDA Center for Biologics Evaluation and Research & U.S. Department of Health and Human Services FDA Center for Devices and Radiological Health Health Qual Life Outcomes (2006) 4, 79. 10.1186/1477-7525-4-79

[9] U.S Department of Health and Human Services Food and Drug Administration Guidance for Industry: Patient-Reported Outcome Measures: Use in Medical Product Development to Support Labeling Claims. U.S. FDA, Clinical/Medical; 2009. http://www.fda.gov/downloads/Drugs/GuidanceComplianceRegulatoryInformation/Guidances/UCM193282.pdf.

[10] Patient-Focused Drug Development. Guidance 2 Discussion Document: Methods to identify what is important to patients. https://www.fda.gov/downloads/Drugs/NewsEvents/UCM620707.pdf. Accessed 31 Jan 2019.

[11] Patient-Focused Drug Development. Guidance 3 Discussion Document: Select, Develop or Modify Fit-for-Purpose Clinical Outcomes Assessments. https://www.fda.gov/downloads/Drugs/NewsEvents/UCM620708.pdf. Accessed 31 Jan 2019.

[12] US Food and Drug Administration. (2018). Patient-Focused Drug Development: collecting comprehensive and representative input. Guidance for industry. Food and Drug Administration staff, and other stakeholders draft guidance.

[13] Critical Path Institute (2015). Interpreting Change in Scores on COA Endpoint Measures [PowerPoint Slides]. Retrieved from https://c-path.org/wp-content/uploads/2015/05/2015_Session6-InterpretingChangeScoresCOAEndpoints.pdf.

[14] Patrick DL, Burke LB, Gwaltney CJ (2011). Content validity—Establishing and reporting the evidence in newly developed patient-reported outcomes (PRO) instruments for medical product evaluation: ISPOR PRO good research practices task force report: Part 1—Eliciting concepts for a new PRO instrument. Value Health.

[15] Morgan, D. L. (1997). Planning and research design for focus groups. Focus Groups as Qualitative Research. Thousand Oaks: SAGE Publications, 32-46.7

[16] Boulkedid R, Abdoul H, Loustau M, Sibony O, Alberti C (2011). Using and reporting the Delphi method for selecting healthcare quality indicators: A systematic review. PLoS One.

[17] Katz NP, Paillard FC, Ekman E (2015). Determining the clinical importance of treatment benefits for interventions for painful orthopedic conditions. J Orthop Surg Res.

[18] Arbuckle, R., Staunton, H., Sully, K., Tomkins, S., Khindri, S., Svedsater, H., & Nelsen, L. (2018). Use of Both Qualitative and Quantitative Methods to Estimate Meaningful Change Thresholds for Key Endpoints in Pediatric Asthma Trials. *Value in Health, *1-8. 10.1016/j.jval.2018.09.2845 30832972

[19] Burbridge C, Hudgens, S., Knight-West, O., Symonds, T. (2017) Optimizing Multiple Raters in the Generation of Anchors for Evaluating Meaningful Change. *Quality of Life Research*, *26* (Suppl 1):4-5

[20] Halling, K. (2016). Frontloading clinical programs with patients’ perspective of meaningful change. Example of GERD. [PowerPoint Slides]. Retrieved from https://c-path.org/wp-content/uploads/2016/01/2016_session6_understandingmeaningfulchange.pdf.

[21] Martin, M., Ortmeier, B. G. (2016). Defining an Outcome Meaningful to Patients. [PowerPoint Slides]. Retrieved from https://onlinelibrary.wiley.com/doi/full/10.1111/jsm.12071.

[22] Orri M, Abraham L, Giraldi A (2013). A phase 2a multicenter, double-blind, placebo-controlled, crossover trial to investigate the efficacy, safety, and toleration of CP-866,087 (a high-affinity mu-opioid receptor antagonist) in premenopausal women diagnosed with female sexual arousal disorder (FSAD). The Journal of Sexual Medicine.

[23] Blome C, Augustin M (2015). Measuring change in quality of life: Bias in prospective and retrospective evaluation. Value in Health.

[24] Willgoss T, Staunton H, Abetz-Webb L, Arbuckle R, Rofail, D. (2017). Qualitative exit interviews with psychologists and parents/caregivers to help inform clinically meaningful change thresholds on an observer-reported clinician administered measure and a performance outcome measure in Down syndrome. *Quality of Life Research*, 26 (Suppl 1):4-5.

[25] Sparrow, S. S. (2011). Vineland adaptive behavior scales. In Encyclopedia of clinical neuropsychology (pp. 2618-2621). Springer, New York.

[26] Christopher Randolph, Michael C. Tierney, Erich Mohr & Thomas N. Chase (1998) The Repeatable Battery for the Assessment of Neuropsychological Status (RBANS): Preliminary Clinical Validity, *Journal of Clinical and Experimental Neuropsychology, 20*(3), 310-319, 10.1076/jcen.20.3.310.823. 10.1076/jcen.20.3.310.823 9845158

[27] US Food and Drug Administration, Center for Drug Evaluation and Research. Xermelo NDA 208794 summary review, February 28, 2017. https://www.accessdata.fda.gov/drugsatfda_docs/nda/2017/208794Orig1s000SumR.pdf. Retrieved 31 Jan 2018

[28] Gelhorn HL, Kulke MH, O’Dorisio T (2016). Patient-reported symptom experiences in patients with carcinoid syndrome after participation in a study of telotristat etiprate: A qualitative interview approach. Clinical Therapeutics.

[29] Ervin CM, Reasner DS, Hanlon JT, Fehnel SE (2017). Exploring the diabetic gastroparesis patient experience: Patient exit interviews. Advances in Therapy.

[30] McGraw S, Qian Y, Henne J, Jarecki J, Hobby K, Yeh W-S (2017). A qualitative study of perceptions of meaningful change in spinal muscular atrophy. BMC Neurology.

[31] Peay H, Kennedy A, Fischer R, Bronson A, Furlong P (2016). Promoting meaningful clinical trial outcome measures for Duchenne muscular dystrophy. Neuromuscular Disorder.

[32] Cook KF, Victorson DE, Cella D, Schalet BD, Miller D (2015). Creating meaningful cut-scores for neuro-QOL measures of fatigue, physical functioning, and sleep disturbance using standard setting with patients and providers. Quality of Life Research.

[33] Cook KF, Kallen MA, Victorson D, Miller D (2015). How much change really matters? Development and comparison of two novel approaches to defining clinically important differences in fatigue scores. Quality of Life Research.

[34] Grant L, Trennery C, Larsen LS, Arbuckle R. (2018). Qualitative methods for exploring meaningful change thresholds, including the use of vignettes: Value, implementation and learnings. *Quality of Life Research, 27*(Suppl 1), 161-162

[35] Wyrwich KW, Fihn SD, Tierney WM, Kroenke K, Babu AN, Wolinsky FD (2003). Clinically important changes in health-related quality of life for patients with chronic obstructive pulmonary disease. Journal of General Internal Medicine.

[36] Bellamy N, Anastassiades T, Buchanan W (1991). Rheumatoid arthritis antirheumatic drug trials. III. Setting the delta for clinical trials of antirheumatic drugs--results of a consensus development (Delphi) exercise. The Journal of Rheumatology.

[37] Bellamy N, Carette S, Ford P (1992). Osteoarthritis antirheumatic drug trials. III. Setting the delta for clinical trials--results of a consensus development (Delphi) exercise. The Journal of Rheumatology.

[38] Revicki D, Hays RD, Cella D, Sloan J (2008). Recommended methods for determining responsiveness and minimally important differences for patient-reported outcomes. Journal of Clinical Epidemiology.

[39] Cella D, Hahn EA, Dineen K (2002). Meaningful change in cancer-specific quality of life scores: Differences between improvement and worsening. Quality of Life Research.

[40] Humphrey L, Willgoss T, Trigg A (2017). A comparison of three methods to generate a conceptual understanding of a disease based on the patients’ perspective. Journal of patient-reported outcomes.

[41] Cappelleri, J. C., Zou, K. H., Bushmakin, A. G., Alvir, J. M. J., Alemayehu, D., & Symonds, T. (2013). *Patient-reported outcomes: Measurement, implementation and interpretation*. Taylor and Francis group.

[42] Cella D, Bullinger M, Scott C, Barofsky I. (2002). Group vs individual approaches to understanding the clinical significance of differences or changes in quality of life. *Mayo Clinic Proceedings, 77*(4), 384-392. 10.4065/77.4.384 11936936

[43] Benjamin K, Vernon MK, Patrick DL, Perfetto E, Nestler-Parr S, Burke L (2017). Patient-reported outcome and observer-reported outcome assessment in rare disease clinical trials: An ISPOR COA emerging good practices task force report. Value in Health.

[44] Creswell, J. W., & Clark, V. L. P. (2007). *Designing and conducting mixed methods research*. SAGE publications.

